# Multisectoral Actions for Health: Challenges and Opportunities in Complex Policy Environments

**DOI:** 10.15171/ijhpm.2017.61

**Published:** 2017-05-16

**Authors:** Viroj Tangcharoensathien, Orapan Srisookwatana, Poldej Pinprateep, Tipicha Posayanonda, Walaiporn Patcharanarumol

**Affiliations:** ^1^International Health Policy Program (IHPP), Ministry of Public Health, Nonthaburi, Thailand.; ^2^National Health Commission Office, Nonthaburi, Thailand.

**Keywords:** Multisectoral Action, Health, Policy, Challenge, Opportunity, Capacity

## Abstract

Multisectoral actions for health, defined as actions undertaken by non-health sectors to protect the health of the population, are essential in the context of inter-linkages between three dimensions of sustainable development: economic, social, and environmental. These multisectoral actions can address the social and economic factors that influence the health of a population at the local, national, and global levels. This editorial identifies the challenges, opportunities and capacity development for effective multisectoral actions for health in a complex policy environment. The root causes of the challenges lie in poor governance such as entrenched political and administrative corruption, widespread clientelism, lack of citizen voice, weak social capital, lack of trust and lack of respect for human rights. This is further complicated by the lack of government effectiveness caused by poor capacity for strong public financial management and low levels of transparency and accountability which leads to corruption. The absence of or rapid changes in government policies, and low salary in relation to living standards result in migration out of qualified staff. Tobacco, alcohol and sugary drink industries are major risk factors for non-communicable diseases (NCDs) and had interfered with health policy through regulatory capture and potential law suits against the government. Opportunities still exist. Some World Health Assembly (WHA) and United Nations General Assembly (UNGA) resolutions are both considered as external driving forces for intersectoral actions for health. In addition, Thailand National Health Assembly under the National Health Act is another tool providing opportunity to form trust among stakeholders from different sectors.

Capacity development at individual, institutional and system level to generate evidence and ensure it is used by multisectoral agencies is as critical as strengthening the health literacy of people and the overall good governance of a country.

The international community recognized that health is a precondition for, an outcome of, and an indicator of all three dimensions of sustainable development — economic, social, and environmental — in a balanced and integrated manner.^1,2^ This has led to the United Nations General Assembly (UNGA) adoption of agenda 2030 for sustainable development where health is enshrined in Goal 3. The Sustainable Development Goals (SDGs) follow, and expand upon, the Millennium Development Goals (MDGs) which expired at the end of 2015, though all health-related MDGs continue to be included in the SDGs with newer targets.

The interlinked nature of the 17 SDGs and 169 targets necessitates effective multisectoral policies and actions. For example addressing poverty, hunger, food insecurity and malnutrition, quality education, employment and decent work are all embraced within an equity framework and interwoven with health considerations. These multisectoral actions synergistically contribute to the health and wellbeing of the population, to economic productivity and the prosperity of a nation.

Multisectoral action for health is defined as actions undertaken by non-health sectors, possibly but not necessarily in collaboration with the health sector, on health or on the determinants of health or health equity. These actions can address the social and economic factors that influence the health of a population at the local, national, and global levels.^3^

This editorial reviews and identifies the challenges of, opportunities and capacity development for effective multisectoral actions for health in a complex policy environment.

## Challenges of Multisectoral Actions for health

In a simple policy environment, relevant sectors may have shared visions where their sectoral goals are mutually gained. Although working across sectors can be challenging, collective multisectoral actions are less complex. For example, in confronting zoonotic diseases which threaten human security such as the H5N1 outbreaks in 2004, it is compelling that wildlife, animal, agriculture and public health agencies felt that their institutional mandates could only be achieved by “working together” to gain a mutual benefit.^4^ In this case, multisectoral action was an enabling tool to facilitate their “mutual gain.”^5,6^

In more complex policy environments, responsibilities are shared across sectors with unclear boundary, or where there might be conflicting sectoral goals and institutional mandates. Here, implementing multisectoral action is the most challenging. For example, to address non-communicable disease (NCD) epidemics, a country needs effective control on tobacco, alcohol and unhealthy food consumption. Such measures as controlling of inappropriate marketing and raising taxes on tobacco, alcohol, unhealthy foods and sugary soft drinks should be implemented. See Box 1 on core interventions, which have been proved as the “best buy” actions against NCD risk factors.^7^

**Box 1. Population-Based “Best Buy” Core Interventions Addressing NCD Risk Factors**

- Tobacco use: Tax increases; smoke-free indoor workplaces and public places; health information and warnings about tobacco; bans on advertising and promotion
- Harmful alcohol use: Tax increases on alcoholic beverages; comprehensive restrictions and bans on alcohol marketing; restrictions on the availability of retailed alcohol
- Unhealthy diet and physical inactivity: Salt reduction through mass media campaigns and reduced salt content in processed foods; replacement of trans-fats with polyunsaturated fats; public awareness programmes about diet and physical activity

Source: Reference 7.

To address the key risk factors of NCD, governments in developing countries always face severe resistance from tobacco, alcohol, food and beverage industries (often transnational) including their proxies, as well as the misinformed scientific community and public media. Their economic, manipulation and political lobbying power in existence should not be underestimated, especially in the context of poor governance in many developing countries. See Box 2 on tobacco industry tactics for resisting public policy on health.^8^ Corporate sponsorships are among the common marketing tools applied.^9^

**Box 2. Tobacco Industry Tactics for Resisting Public Policy on Health**

The tobacco industries’ tactics include conducting public relations campaigns, buying scientific and other expertise to create controversy about established facts, funding political parties, hiring lobbyists to influence policy, using front groups and allied industries to oppose tobacco control measures, pre-empting strong legislation by pressing for the adoption of voluntary codes or weaker laws, and corrupting public officials.

Source: Reference 8.

Legal threats or filing law suits against governments are common practices by the tobacco industry, for example, when the government introduced graphic health warnings on cigarette packages in Thailand,^10^ and plain cigarette packaging in the United Kingdom.^11^

Philip Morris Australia filed a lawsuit against the Australian government on the grounds that “plain packaging violated the Australian Constitution, because the government seeks to acquire its property without paying compensation.” With strong evidence that plain packaging can reduce positive perceptions of smoking and dissuade tobacco use, Australia won the legal battle with Philip Morris over plain packaging.^12^

A broad international consensus among researchers shows that the most effective measures to address problems caused by alcohol are to raise the price, control availability and restrict marketing activities. However, a study from the United Kingdom^13^ shows that the alcohol industry constructed doubt about this wealth of scientific evidence and instead chose to promote weak survey-based evidence and made unsubstantiated claims to their advantage.

There is no evidence of a threshold for the carcinogenic effects of asbestos; increased cancer risks have been observed in populations exposed to very low levels.^14,15^ A total ban on the use of all types of asbestos^16^ recommended by the World Health Organization (WHO) is supported by International Labour Organisation Resolution of 2006.^17^ This promotes the elimination of future use of all forms of asbestos, in order to protect the health of the workers and consumers.

The Thailand National Health Assembly adopted in 2010 a resolution for the total ban of chrysotile asbestos, which was further endorsed by a Cabinet Resolution. Resistance by the industry was evident, and it falsified information to the public through the media. Also, a government agency in favour of the industry challenged the validity of WHO evidence and commissioned an academic institute^18^ to do research. The institute then recommended further study on the health implications of chrysotile, improved safe use of chrysotile and increased public awareness on safe use. Furthermore, its report highlighted the concerns of the economic impact, if Thailand applies total ban on import and export, the industry will lose from not able to export chrysotile-containing materials to other countries in the Association of Southeast Asian Nations where there is no ban (for importation) and the large social cost of replacement by alternate materials.

To address the lead killer of road traffic injuries, there needs to be effective enforcement on drink-driving, speed limits and the use of helmets. This should come with strong support from the police, local government and active citizens against the challenges masterminded by the strong and effective marketing on alcoholic beverages. Fatal traffic collisions among drunk drivers even at the legal limits of 0.05 mg/mL blood alcohol concentration (BAC) are commonly reported, because impairment in critical driving functions begins at very low BAC levels.^19^ Despite ample evidence, government efforts to further reduce the 0.05 mg/mL limits in most countries were hampered by alcohol lobbyists and media proxies, despite the evidence.^20^

*“An attempt in New Zealand to lower the legal BAC for driving to 0.05 mg/mL in late 2003 (from 0.08 mg/mL) was not supported at Cabinet. Media analysis of the lead-up to the Cabinet decision not to pass the 0.05 level shows that policy is unlikely to be adopted in the face of ambivalence and lack of cohesion on the part of the public health sector and a strong media representation of the industry position that ‘the proposal is incomprehensible when the majority of New Zealanders drive responsibly and keep under the limit.’”* ^21^

Brazil successfully reduced the legal BAC limits from 0.06 to 0.02 mg/mL in 2008. The impact was significant and greater on traffic fatalities than on injuries, and higher effects were observed in the capital city where police enforcement was stronger.^22^

## Opportunities for Multisectoral Actions for Health

Despite these challenges, opportunities exist for multisectoral actions to ensure that the people’s health is protected by public or private policies. Several external driving forces encourage multisectoral actions for health. For example, all these demand effective multisectoral actions for health: resolutions by the World Health Assembly, Food and Agriculture Organization and the International Organization of Animal Health to tackle anti-microbial resistance (AMR) based on the One Health approach, the Framework Convention on Tobacco Control, the International Health Regulation, the UN high level Declaration on NCD, the UN Convention on Rights of People with Disability, various resolution adopted by the UNGA and World Health Assembly such as on universal health coverage, and the SDGs.

Thailand has enacted a National Health Act since 2007, which establishes a National Health Commission, chaired by the Prime Minister. The commission members comprise one-third from the multisectoral public policy-makers; one-third from academia and professionals; and one-third from civil society organizations including private sector. This tri-partite constituency applies the concept of the “triangle that moves the mountain”^23^ through convening an annual National Health Assembly, as mandated by Law. The Assembly adopted several landmark resolutions, in particular those requiring multisectoral actions for health.

The National Health Assembly has shown some success on multisectoral action for health.^24^ Bringing in all key actors including the private sector, who are part of the “problem” and part of the “solution” to the National Assembly Process is beneficial. In front of the public and under the spotlight, gradually, every actor including the private sector can learn about real “societal responsibilities” and moderates their business model to respond to well-informed and empowered consumers.

The National Health Assembly has proved that trust among stakeholders forms the basis of effective multisectoral actions for health, also confirmed by the statement:

*“Building and nurturing trust among all partners has been a challenging and time-consuming task in many countries, but it has also ensured a strong foundation for effective working relationships*.” ^3^

The execution of soft power through the National Health Assembly also runs parallel with the rule of law and legal enforcement for violations such as those committed by the tobacco and alcohol industries. A good balance between soft power and legal actions is essentially an innovative model of the application of multisectoral action for health.

## Capacities and Capacity Development Required for Multisectoral Actions for Health

Capacity is defined as the ability of people, organizations and society to manage their affairs successfully; while capacity development is the process whereby people, organizations and society unleash, strengthen, create, adapt and maintain capacity over time.^25^ Capacity is also defined as skills, knowledge, resources needed to perform a function, while capacity development is the process by which individuals, groups, organizations, institutions and countries develop their abilities, individually and collectively, to perform functions, solve problems and achieve objectives.^26^

Capacity building is the process aiming to facilitate, in conjunction with the stakeholders, a consolidation of their capacities at an individual, organizational and sectoral level to allow them to evolve and adapt to the new contextual requirements and fulfill their role within a governance structure.^27^

The key barriers to developing the capacity of the public sector are two folds.^25^ The first is poor governance such as entrenched political and administrative corruption, widespread clientelism, unclear and arbitrary enforcement of rules of law, lack of effective citizen voice, weak social capital, lack of trust and respect for human rights.

The second is lack of government effectiveness such as poor capacity to undertake strong public financial management, and low levels of transparency and accountability leading to rampant corruption. It is also about rapid changes in government policies, unpredictable and inflexible funding and staffing, and low salaries in relation to living standards. This results in the migration out of qualified staff, excessive reliance on donor-funded positions and a lack of rewards for performance and of sanctions for non-performance.

These common barriers should be identified based on each country’s political and cultural context. It is important to prioritize them and gradually address them.

A few key capacities at individual, institutional and system levels are needed for multisectoral actions for health. Health literacy of individual and community is essential and must be strengthened. Individual and institutional capacities are needed to generate evidence on positive and negative health implications of certain public or private sectoral policies. The evidence should be translated into multisectoral policy decisions through a transparent process of participation and engagement by relevant stakeholders including government, citizen and private sectors. Progress should be monitored through regular reports which are publicly available.

Effective communication and storytelling to the public will gradually help form public opinion and social consensus for which politicians are sensitive to public opinion. It is good to seize opportunities every cycle of a general election in order to form political manifestos and commitment towards improving the health of the population. For example, policy champions contributed significantly to framing tobacco policy and legislation in the United States^28^ and Thailand^29^ and the role of civil society in fighting corruption has been recognized by the UN Convention Against Corruption in articles 5, 13, and 63 (4) (c).^30^

Capacities developed in independent or quasi-independent think-tank agencies^31,32^ have led to greater positive outcomes and are more sustainable than in government agencies. There is a high turnover rate of professionals in government agencies and it is difficult to sustain capacities in the longer term. Capacity development for health policy and systems research is as important as sustaining capacities in a supportive environment which enriches people’s contributions.^33^

At a system-wide level, there is a real need to strengthen good governance, in particular the rule of law to ensure the following: transparency, that the voice of citizen is heard, and ethical leadership. There should be zero tolerance to corruption, regulatory capture, a form of government failure, where the regulatory agency fails to act for the public interest^34^ and state capture where firms are able to shape the laws, policies, and regulations of the state to their own advantage by providing illicit private gains to public officials.^35^

Effective multisectoral actions for health require consensus across all partners to reach a “shared vision.”^36^ Shared vision can be perceived as a common ground where each institutional vision lies within. The shared vision is based on trust and respect; but the risk is that it can be undermined. Nevertheless we must strive together, and work to overcome the challenges we face in taking multisectoral actions to meet the great health needs of the 21st century.

## Conclusion

Despite challenges in effective multisectoral actions for health; certain opportunities arise where key actions should be taken.

Actors across ministries need to reach consensus on a shared vision where they contribute concertedly to the common health goals. Good governance in government agencies and private enterprises is the key contributors to multisectoral actions for health. Active citizenship and participation in the deliberative democracy to form social consensus on certain public or private policies which have implications on health of the people, had gradually emerged in developing countries; such as Thailand National Health Assembly. Government needs to strengthen and sustain capacities to fight against law suits commonly used by industries such as tobacco, alcohol, sugary beverage and unhealthy food. The direct command and control such as ensure smoke free public spaces and restrict availability of alcohol are as important as policies to increase tax and retail prices of these products.

Zero tolerance to corruption can be gradually achieved through stringent law and enforcement where both legal and social sanctions are functioning including active citizenship, strong civil society organizations. Other social movements toward good governance are required in both public sector and private enterprises, which include such as rule of law, voice and accountability, and government effectiveness.

## Ethical issues

Not applicable.

## Competing interests

Authors declare that they have no competing interests.

## Authors’ contributions

VT led the discussion and developed the structure of this article together with other authors. TP and WP searched relevant literatures. OS, PP, TP, and WP reviewed literatures. VT, OS, and PP synthesized the article together with TP and WP. All authors read and approved the final article.

## Authors’ affiliations

^1^International Health Policy Program (IHPP), Ministry of Public Health, Nonthaburi, Thailand. ^2^National Health Commission Office, Nonthaburi, Thailand.
